# Supplementary material for: Cardiac Rehabilitation for Older Women with Heart Failure
Source: J Pers Med. 2022 Nov 30;12(12):1980. doi: 10.3390/jpm12121980 (PMC9785443; doi:10.3390/jpm12121980)
Supplement: Supplementary file 1 [file jpm-12-01980-s001.zip › jpm-2034495-SI.pdf]

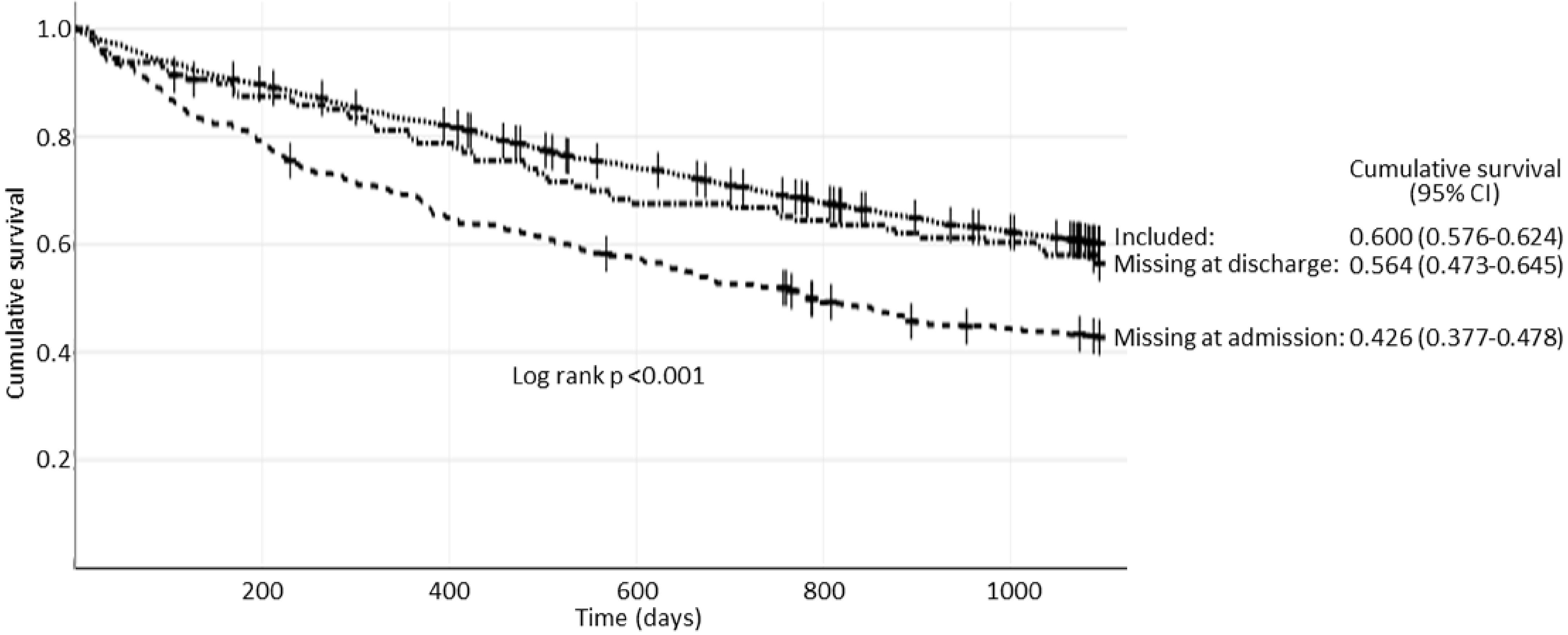

Figure S1. Survival curves for patients with missing data for 6-min walk test at admission or at discharge, compared with included patients.
